# Genetically Predicted Longer Telomere Length May Reduce Risk of Hip Osteoarthritis

**DOI:** 10.3389/fgene.2021.718890

**Published:** 2021-10-05

**Authors:** Jing Yang, Huiqing Xu, Bingyue Cai, Jiahe Wei, Lingling Sun, Yasong Li, Tianle Wang, Yingjun Li

**Affiliations:** ^1^ Zhuji People’s Hospital of Zhejiang Province, Zhuji Affiliated Hospital of Shaoxing University, Zhuji, China; ^2^ School of Public Health, Hangzhou Medical College, Hangzhou, China; ^3^ Department of Orthopaedics, The Second Affiliated Hospital, School of Medicine, Zhejiang University, Hangzhou, China; ^4^ Department of Rheumatology and Immunology, Zhejiang Provincial People’s Hospital, People’s Hospital of Hangzhou Medical College, Hangzhou, China

**Keywords:** hip OA, total OA, telomere length, mendelian randomization, knee OA

## Abstract

**Objective:** This two-sample Mendelian randomization (MR) study aimed to examine the potential causal association of telomere length (TL) with the risk of osteoarthritis (OA).

**Method:** The summary-level data for OA was derived from the United Kingdom Biobank cohort, including 50,508 individuals of European descent. Eighteen single nucleotide polymorphisms associated with TL were identified as instrumental variables from the most up-to-date TL genome-wide association study (GWAS) involving over 78,592 individuals of European descent. Based on the GWASs data, MR was performed using established statistical analysis methods including the inverse variance weighted, weighted median, MR-Egger, and MR pleiotropy residual sum and outlier.

**Results:** Genetically determined TL was not associated with the risk of total OA (IVW odds ratio [OR] = 1.00, 95% confidence interval [CI] = 0.83, 1.21). In subgroup analyses stratified by OA site, no evidence in favor of association between genetically determined TL and knee OA was found (IVW OR = 1.18, 95% CI = 0.89, 1.58). However, using WM method, we observed a limited protective effect of longer TL on the risk of hip OA (OR = 0.60, 95% CI = 0.36–0.99), whereas the results of the IVW (*p* = 0.931) and MR-PRESSO (*p =* 0.932) showed that TL had no effect on hip OA.

**Conclusions:** This study does not support a causal association between TL and total OA. A potential protective association between longer TL and hip OA, though possible, remains less certain.

## Introduction

Osteoarthritis (OA) is a chronic disease that causes joint-specific pains and disabilities (Glyn-Jones et al., 2015). The latest data shows that over 303.1 million people are under the burden of increasing medical expenses and a declining quality of life produced by OA (Peat and Thomas, 2021), which involves any joint including hands, hips and knees, leading to irreversible cartilage loss or bone sclerosis (Kalamegam et al., 2018). It’s well accepted that the incidence of OA has a close relationship with aging and other risk factors (Sacitharan, 2019).

Telomeres are TTAGGG repeats bound by associated protein complexes that are located at the end of chromosomes to maintain genome stability (Loe et al., 2020). Many studies have confirmed that telomeric repeats shorten with each cell division (Martinez and Blasco, 2018). Therefore, telomere length (TL) is often used as an indicator of aging. Recently, the association between telomeres and multiple age-related diseases including OA has been constantly reported (Martin and Buckwalter, 2001; Michou, 2011; Ahmed et al., 2018). However, the nature and causality of such relationship remain inconclusive. For example, a few cross-sectional studies showed that patients with OA had shorter telomeres than normal controls (Tamayo et al., 2011; Fellows et al., 2017; McAlindon et al., 2018). However, a case-control study done by Rose et al. showed that only genomic DNA damage of a higher degree occurred in OA, but the corresponding telomeres did not shorten after comparing OA cartilage and normal cartilage through autopsies (Rose et al., 2012). In addition, traditional epidemiological studies are prone to bias due to confounding factors and reverse causality, which, to a certain extent, are limited by the study sample size (Larsson et al., 2019); therefore, the causal relationship between TL and the risk of OA cannot be reliably inferred based on the observational results.

Mendelian randomization (MR) is a novel method that follows the law of independent assortment, in which genetic variants are used as instrumental variables (IVs) to assess the causal effect of exposure on outcome (Burgess et al., 2017b; Bowden and Holmes, 2019). Since the genotype of an individual is determined during conception and cannot be changed, the reverse causality between the genetic phenotype and the associated outcome is largely avoided through this method. Furthermore, based on the publicly available databases, the causal relationship between exposure and outcome can be inferred more economically and efficiently through this design.

As far as we know, no MR analysis assessing the causal association between TL and OA has been carried out by now. Here, we performed a two-sample MR study to clarify whether there existed a causal relationship between TL and the risk of OA.

## Materials and Methods

### Data Sources

This study was based on summary data from a genome-wide association study (GWAS) of OA, which contained across 16.5 million variants from the resources of United Kingdom Biobank. The GWAS comprised 10,083 cases and 40,425 controls of predominantly European descent (Zengini et al., 2018). In addition, to investigate the causal link between TL and site-specific OA, summary statistics from the GWAS for knee and hip OA, which were two major subgroups of OA, were added into the study. We defined OA cases based on diagnosis records of hospitals. The diagnosis of OA coding by a hospital in the United Kingdom Biobank was based on the ICD-10 code captured from the data of Hospital Episode Statistics (HES). The sample characteristics in this study were described in Supplementary Table S1.

A hitherto largest genome-wide meta-analysis on leukocyte TL was conducted among up to 78,592 individuals of European descent, in which TL was measured through an established quantitative PCR technique and expressed as a ratio of the number of telomere repeats to a single-copy gene (Li et al., 2020). In the meta-analysis, a standardized quality control (QC) for exclusion [random-effect meta-analysis on single nucleotide polymorphisms (SNPs) with a Cochrane’s Q *p* value <1 × 10^–6^, a minor allele frequency ≥1% and a sample size ≥40%] has been implemented, resulting in a total number of 2,362,330 SNPs. After adjusting the age, gender and cohort-specific covariates, Li et al. identified 20 SNPs associated with TL at genome-wide association significance (*p* < 5 × 10^–8^).

All participants provided a written informed consent, which was included in the study together with their aggregated data to be used for scientific publications.

### SNP Selection

**FIGURE 1:**
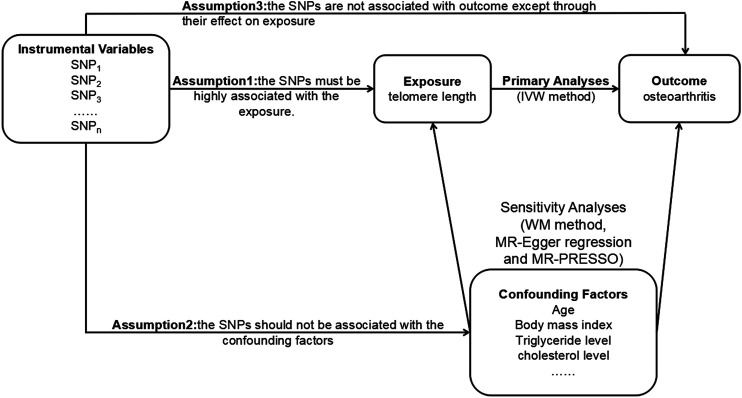
The principles of the MR method. MR, Mendelian randomization; SNP, single nucleotide polymorphism; IVW, inverse variance weighted; WM, weighted median; MR-PRESSO, MR pleiotropy residual sum and outlier.

The MR method relies on three core assumptions (Figure 1), which are as follows: the SNPs must be highly associated with the exposure (the relevance assumption); they should not be associated with the confounding factors (the independence assumption); they are not associated with outcome except through their effect on exposure (the exclusion restriction assumption) (Burgess et al., 2017b). SNPs can be treated as IVs only when the three assumptions are met.

In our MR analysis, a linkage disequilibrium (LD) test was performed on each SNP identified as a genetic instrument. The LD threshold used for pruning was 0.05. And one SNPs (rs2853677) was removed at *r* ^2^ > 0.05 and kb < 5,000. We replaced the specific SNPs not identified in OA GWAS with the proxy in high LD (*r* ^2^ > 0.8). In addition, we did not exclude palindromic SNPs at MAF <0.3.

### Statistical Analysis

In this study, TwoSampleMR and MRPRESSO packages with R software were used to analyze the summarized data (Yavorska and Burgess, 2017). The inverse-variance-weighted (IVW) method was our main MR analysis methodology, through which the causal effect of genetically predicted exposure on related outcome can be estimated (Hartwig et al., 2016). Several sensitivity analyses were carried out through methods including the weighted median (WM) method (Burgess et al., 2017a), the MR-Egger regression method and the MR pleiotropy residual sum and outlier (MR-PRESSO) method (Burgess and Thompson, 2017). To be more specific, the WM estimator can be used to overcome some shortcomings of IVW and gives a uniform estimate of the causal effect even when the invalid instrumental variables accounted for 50% (Bowden et al., 2016). The MR-Egger method is a statistical method through which the estimates are actually robust to horizontal pleiotropy and can reflect causal effect (Bowden et al., 2015). Nonetheless, the estimates through MR-Egger may be inaccurate and can be disturbed by outlying genetic variants. Thus, we corrected for outliers through MR-PRESSO. All statistical analyses were two-sided and considered statistically significant at a *p* value <0.05. Besides, through leave-one-out analysis by removing every single SNP at turn, the reliability of the results could be made sure. The related R scripts were shown in Supplementary Text S1.

In order to reduce the effect of covariates (i.e., confounders) on causality assessments, we manually screened the SNPs by using the Phenoscanner database and excluded the those associated with confounders. Furthermore, the strength of the IVs was assessed by calculating *R* ^*2*^ and the *F*-statistics (Burgess and Thompson, 2011).

## Results

SNPs associated with TL of European ancestry are presented in Table 1 (Zengini et al., 2018). All of them are associated with TL at a level of genome-wide statistical significance (*p* < 5 × 10^–8^). After searching them in Phenoscanner database, we found that only rs73624724 (*RTEL1*) showed an association with confounding factors (e.g., body and leg fat percentage). We performed an analysis excluding this SNP. Besides, to verify whether the SNPs met Assumption 3, we analyzed the association between the SNPs and the outcome. No SNP was significantly associated with OA (*p*-value was not much lower than 0.05). Lastly, 18 sentinel variants at 17 genomic loci were taken as IVs for TL. The *F*-statistics of all SNPs were above the threshold of 10, ensuring that they were strong instruments.

**TABLE 1:** Characteristics of SNPs used as genetic instruments for TL in the present MR study.

SNP	Chr	Position	Nearest gene	Effect allele	Non-effect allele	EAF	TL			OA			*R* ^*2*^ ^a^	*F*-statistic^b^	Remove
							Beta	SE	*p* Value	Beta	SE	*p* Value			
rs3219104	1	226562621	PARP1	C	A	0.83	0.042	0.006	9.60E-11	−0.033	0.022	0.13	0.0006	49	-
rs10936600	3	169,514,585	TERC	T	A	0.24	−0.086	0.006	7.18E-51	0.006	0.018	0.74	0.0026	205	-
rs4691895	4	164,048,199	NAF1	C	G	0.78	0.058	0.006	1.58E-21	−0.022	0.019	0.24	0.0012	93	-
rs7705526	5	1,285,974	TERT	A	C	0.33	0.082	0.006	5.34E-45	0.008	0.017	0.66	0.0024	187	-
rs2853677	5	1,287,194	TERT	A	G	0.59	−0.064	0.006	3.35E-31	−0.007	0.016	0.65	0.0014	114	*r* ^2^ > 0.05^c^
rs59294613	7	124,554,267	POT1	A	C	0.29	−0.041	0.006	1.17E-13	−0.007	0.017	0.69	0.0006	47	-
rs9419958	10	105,675,946	STN1	C	T	0.86	−0.064	0.007	5.05E-19	0.005	0.023	0.84	0.0011	84	-
rs228595	11	108,105,593	ATM	A	G	0.42	−0.029	0.005	1.43E-08	0.004	0.016	0.81	0.0004	34	-
rs2302588	14	73,404,752	DCAF4	C	G	0.10	0.048	0.008	1.68E-08	0.024	0.026	0.36	0.0005	36	-
rs7194734	16	82,199,980	STMN3	T	C	0.78	−0.037	0.006	6.94E-10	−0.024	0.019	0.19	0.0005	38	-
rs8105767	19	22,215,441	ZNF208	G	A	0.30	0.039	0.005	5.42E-13	0.007	0.017	0.67	0.0008	61	-
rs75691080	20	62,269,750	STMN3	T	C	0.09	−0.067	0.009	5.88E-14	−0.026	0.029	0.37	0.0007	55	-
rs34978822	20	62,291,599	RTEL1	G	C	0.02	−0.140	0.023	7.26E-10	0.104	0.053	0.05	0.0005	37	-
rs73624724	20	62,436,398	RTEL1	C	T	0.13	0.051	0.007	6.33E-12	−0.028	0.023	0.22	0.0007	53	body fat percentage^d^
rs55749605	3	0,1,232,093	SENP7	A	C	0.58	−0.037	0.007	2.45E-08	−0.009	0.016	0.56	0.0004	28	-
rs13137667	4	71,774,347	MOB1B	C	T	0.96	0.077	0.014	2.43E-08	0.036	0.049	0.46	0.0004	30	-
rs34991172	6	25,480,328	CARMIL	G	T	0.07	−0.061	0.011	6.19E-09	0.031	0.028	0.28	0.0004	31	-
rs2736176	6	31,587,561	PRRC2A	C	G	0.31	0.035	0.006	3.53E-10	−0.006	0.017	0.73	0.0004	34	-
rs3785074	16	69,406,986	TERF2	G	A	0.26	0.035	0.006	4.64E-10	0.004	0.017	0.83	0.0004	34	-
rs62053580	16	74,680,074	RFWD3	G	A	0.17	−0.039	0.007	4.08E-08	−0.050	0.021	0.02	0.0004	31	-

Abbreviation: SNP, single nucleotide polymorphism; Chr, chromosome; EAF, effect allele frequency; TL, telomere length; SE, standard error; OA, osteoarthritis.

a *R* ^*2*^ was calculated using the following formula: (2×EAF×(1-EAF)×Beta^2^)/[(2×EAF×(1-EAF)×Beta^2^) + (2×EAF×(1-EAF)×N×SE^2^)], where Beta is the estimated effect on telomere length, *Ν* is the sample size of the GWAS for the SNP-telomere length association and SE is the standard error of the estimated effect.

b *F* statistic was calculated using the following formula: *R* ^*2*^(N-2)/(1-*R* ^*2*^), where *R* ^*2*^ is the proportion of variance in telomere length explained by each instrument and N is the sample size of the GWAS for the SNP-telomere length association.

c SNP with *r* ^2^ > 0.05 was removed.

d SNP associated with confounding factors (e.g., body fat percentage) was removed.

As is shown in Table 2, the odds ratio (OR) of hip OA per standard deviation (SD) increasing in TL was 0.60 (95% confidence interval [CI] = 0.36, 0.99, *p* = 0.049] through the WM method, while nonsignificant effect estimates were shown through the IVW and MR-PRESSO method. Remarkably, our results showed evidences of weak horizontal pleiotropy (*P* for intercept = 0.050) without heterogeneity (Q = 25.18, *p* = 0.091). Similar results were suggested through MR-Egger adjusting for horizontal pleiotropy based on WM model (OR = 0.34, 95% CI = 0.12, 1.01, *p* = 0.071). Furthermore, genetically predicted TL was not related to the total risks of OA (IVW: OR = 1.00, 95% CI = 0.83, 1.21, *p* = 0.989) and knee OA (IVW: OR = 1.18, 95% CI = 0.89, 1.58, *p* = 0.250), respectively. Similar results were yielded through the WM, MR-Egger and MR-PRESSO method. The MR-Egger analysis suggested that the directional pleiotropy was unlikely to bias the causal effect of TL on total OA and knee OA (*P* for intercept = 0.196 for total OA and *P* for intercept = 0.476 for knee OA). No heterogeneity was detected by Cochran’s Q statistic (*p* = 0.318 for total OA and *p* = 0.273 for knee OA). Scatter plots, forest plots and funnel plots of the total OA, knee OA and hip OA are presented in Supplementary Figure S1–S9.

**TABLE 2:** MR results of the association between TL and OA (18 SNPs).

MR method	OR (95%CI)	*p*-value for association^a^	Cochran’s Q statistic	*p*-value for heterogeneity	*p*-value for MR egger intercept
OA (10,083/40,425)					
IVW	1.00 (0.83, 1.21)	0.989	19.18	0.318	-
WM	1.01 (0.78, 1.30)	0.948	-	-	-
MR-Egger	0.73 (0.45, 1.20)	0.230	-	-	0.196
MR-PRESSO	1.00 (0.81, 1.19)	0.989	-	-	-
Knee OA (4,462/17,885)					
IVW	1.18 (0.89, 1.58)	0.250	20.02	0.273	-
WM	1.19 (0.81, 1.75)	0.366	-	-	-
MR-Egger	0.90 (0.41, 1.98)	0.800	-	-	0.476
MR-PRESSO	1.18 (0.90, 1.47)	0.266	-	-	-
Hip OA (2,396/9,593)					
IVW	1.02 (0.66, 1.59)	0.931	25.18	0.091	-
WM	0.60 (0.36, 0.99)	**0.049**	-	-	-
MR-Egger	0.34 (0.12, 1.01)	**0.071**	-	-	0.050
MR-PRESSO	1.02 (0.58, 1.46)	0.932	-	-	-

Abbreviation: MR, Mendelian randomization; OR, odds ratio; CI, confidence interval; OA, osteoarthritis; IVW, inverse variance weighted; WM, weighted median; MR-PRESSO, Mendelian randomization pleiotropy residual sum and outlier.

a Bolded *P* represents limited significance.

The results of leave-one-out sensitivity analysis showed that no single SNP had a significant effect on the pooled results, suggesting the stability of our results (Supplementary Figure S10–S12).

## Discussion

To the best of knowledge, this is the first MR study examining the relationship between TL and the risk of total OA, as well as OA at specific joint sites (knee and hip). These results suggest that the association between TL and total OA risk is not likely to be causal. Through subgroup analyses, we find some evidences supporting the causal association between TL and hip OA instead of knee OA.

It should be noted that the genetically predicted TL is not associated with the risk of total OA in our MR study. The present study is in disagreement with quite a few previous observational studies. The findings of some case-control studies show that TL is inversely related to the risk of OA (Zhai et al., 2006; Poonpet et al., 2018; Manoy et al., 2020). Similarly, a recently published meta-analysis including ten studies indicates that TL might be a potential biomarker of OA (Xie et al., 2021). On the contrary, as is suggested in another study, there is no association between TL and OA after adjusting age (Tamayo et al., 2010). Taken together, the results of previous epidemiological studies on OA remain contradictory, which may be caused by differences in the joint site, disease progression, population selection or techniques used in the TL measurement. To compute unmeasured confounding in previous observational studies, we reported the E-value, defined as the minimum strength of association on the risk ratio scale that an unmeasured confounder would need to have with both the exposure and the outcome, conditional on the measured covariates, to fully explain away a specific exposure-outcome association (VanderWeele and Ding, 2017). E-value was computed online (https://www.evalue-calculator.com/). The lowest possible E-value is 1. The larger the E-value is, the stronger the unmeasured confounder associations would have to be considered to explain away an effect. More details were shown in Supplementary Table S2.

In the MR study on the OA subgroup, we found that TL was related to the risk of hip OA rather than that of knee OA, thus revealing the specificity at joint sites. For hip OA, our results are largely consistent with that of previous observational studies. An indirect association between TL and the risk of hip OA is reported in a study, supporting the conclusion that telomere shortening is related to cartilage degradation (Harbo et al., 2013). A single or a few critically short telomeres are enough to trigger cellular senescence of normal cells, which result in many degenerative and aging-related diseases including OA. The severity of OA is associated with the increase in the number of senescent cells in joint tissues, as the accumulation of senescent chondrocytes will reduce the ability of chondrocytes to maintain and repair cartilage, so that tissues are unable to bear stress (Harbo et al., 2013). Besides, due to the specific role of the hip joint and specific lifestyles, shorter telomeres are less resistant to inflammation and oxidative stress (OXS), leading to an increased formation of oxLDL particles and a decreased level of spermidine, thus resulting in the development and progression of hip pathology (Tootsi et al., 2017). The above may be potential mechanisms that could explain different associations between TL and specific joint sites.

The causal association between longer TL and hip OA, though possible, remains less certain. Both WM and MR-Egger regression method suggested a limited causal association between TL and hip OA, and the IVW and MR-PRESSO method suggested a null causal association. As far as we know, the WM gives a correct estimate of the causal effect as long as at least 50% of the weight comes from effective IVs. Moreover, compared to IVW, it has better finite-sample Type 1 error rates (Bowden et al., 2016). As for the MR-PRESSO, it detects and corrects the outliers in IVW linear regression. In the study, no outliers were highlighted by MR-PRESSO. The MR-Egger regression method may provide estimates for the true causal effect that is consistent even if all the genetic variants are invalid instruments, as long as the instrument strength independent of direct effects (InSIDE) assumption is satisfied (Bowden et al., 2015). Thus, the effect of TL on hip OA was potentially causal, as indicated by the WM and MR-Egger approach.

The primary strength of MR study is less susceptible to confounding and reverse causality, thereby overcoming the defects of observational studies on the association between TL and OA, which provides a strong support to our result (Fan et al., 2020). There are also several limitations. Firstly, only European individuals participated and were included in this study. Thus, our results might not be generalizable to ancestries other than European, and it is essential to verify our results in other populations. Secondly, only summary-level data was used in the MR analysis, which was not sufficient to make further stratified analyses on other specific factors, such as age and gender, etc. Thirdly, not all MR models showed a statistically significant association between TL and the risk of hip OA. Nevertheless, consistent causal estimates could be provided through the MR-Egger method while taking into account directional pleiotropy. Besides, we conducted an additional sensitivity analysis on potential horizontal pleiotropy, providing evidence for the robustness of our results. Finally, further MR studies as well as longitudinal studies are needed to investigate the association between TL and OA.

In summary, this MR study does not support the causal association between TL and the total OA or knee OA. A potential protective association between longer TL and hip OA, though possible, remains less certain.

## Data Availability

The original contributions presented in the study are included in the article/Supplementary Material, further inquiries can be directed to the corresponding author.
