# Supplementary material for: Genetically Predicted Longer Telomere Length May Reduce Risk of Hip Osteoarthritis
Source: Front Genet. 2021 Oct 5;12:718890. doi: 10.3389/fgene.2021.718890 (PMC8523818; doi:10.3389/fgene.2021.718890)

| Supplementary Table S1 Baseline characteristics of the study population | | | | | | |
| --- | --- | --- | --- | --- | --- | --- |
| Variables | Hospital diagnosed OA | | Hospital diagnosed hip OA | | Hospital diagnosed knee OA | |
|  | Cases | Controls | Cases | Controls | Cases | Controls |
| N | 10,083 | 40,425 | 2,396 | 9,593 | 4,462 | 17,885 |
| Female, N (%) | 5,461 (54.2) | 19,261 (47.6) | 1,301 (54.3) | 4,334 (45.2) | 2,122 (47.6) | 8,073 (45.1) |
| Male, N (%) | 4,622 (45.8) | 21,164 (52.4) | 1,095 (45.7) | 5,259 (54.8) | 2,340 (52.4) | 9,812 (54.9) |
| Mean age at recruitment in years (±SD) | 60.65 (±6.49) | 64.68 (±2.53) | 61.99 (±5.79) | 68.19 (±0.87) | 60.41 (±6.64) | 67.10 (±1.41) |
| Mean age at recruitment in years (±SD) | 29.64 (±5.43) | 27.28 (±4.34) | 29.14 (±5.11) | 27.21 (±4.19) | 30.49 (±5.48) | 27.28 (±4.24) |
| Abbreviation: OA, osteoarthritis; N, number; SD, standard deviation. | | | | | | |

| Supplementary Table S2 Observational study about the association between TL and OA | | | | | | |
| --- | --- | --- | --- | --- | --- | --- |
| PMID | Year | Title | Category | Sample size, cases; controls | Findings | E-value ^a^ |
| 21291897 | 2011 | Decreased length of telomeric DNA sequences and increased numerical chromosome aberrations in human osteoarthritic chondrocytes. | Case-control study | 39; 20 | Shorter TL in OA patients (T/S ratio: 1.64±0.41vs1.99±0.54) | - |
| 28150695 | 2017 | Characterisation of a divergent progenitor cell sub-populations in human osteoarthritic cartilage: the role of telomere erosion and replicative senescence. | Vitro tests | 7; 6 | Shorter TL in OA patients (T/S ratio: 0.73 ± 0.07 vs 1.18 ± 0.04) | - |
| 30172836 ^b^ | 2018 | Incident hand OA is strongly associated with reduced peripheral blood leukocyte telomere length. | Cross-sectional study | 4674; 122 | Shorter TL in OA patients (relative risk= 1.10; 95% CI: 0.96-1.27) | 1.43 |
| 17038452 | 2006 | Reduction of leucocyte telomere length in radiographic hand osteoarthritis: a population-based study. | Cross-sectional study | 160; 926 | Estimate effect in cases (indicated by Total Kellgren/Lawrence score): −0.86 | 4.16 |
| 28205383 | 2018 | Association between leukocyte telomere length and angiogenic cytokines in knee osteoarthritis. | Case-control study | 80; 60 | Shorter TL in OA patients (T/S ratio: 1.1±0.4 vs 1.3±0.6) | - |
| 32765866 | 2020 | Telomere shortening is associated with poor physical performance in knee osteoarthritis. | Case-control study | 202; 60 | Shorter TL in OA patients (T/S ratio: 0.59±0.10 vs 1.31±0.30) | - |
| 34313963 | 2021 | Telomere length in patients with osteoarthritis: a systematic review and meta-analysis. | Meta-analysis | 678; 1457 | Standard mean difference in cases: - 0.32; 95% CI - 0.57 to - 0.06 | 2.01 |
| 22171676 | 2011 | mtDNA haplogroup J modulates telomere length and nitric oxide production. | Case-control study | 79; 166 | Shorter TL in OA patients (T/S ratio: 0.986±0.261 vs 1.008±0.287) | - |
| 19879280 | 2010 | Differing patterns of peripheral blood leukocyte telomere length in rheumatologic diseases. | Case-control study | 34; 130 | No differences | - |

^a^ E-value could not be calculated for some studies due to lack of point estimate and standard error.

^b^ A longitudinal study

Supplementary Text S1 Edited R script used for data analysis

# TwoSampleMR

# Gibran Hemani, Philip Haycock, Jie Zheng, Tom Gaunt, Ben Elsworth, Tom Palmer

# Web source: https://mrcieu.github.io/TwoSampleMR/articles/index.html

#

# Edited by: Huiqing Xu

# August 2021

#Take total OA as an example:

library(TwoSampleMR)

exp_dat <- read_exposure_data(

filename = "TLexposure.txt",

clump = FALSE,

sep= " ",

snp_col = "SNP",

beta_col = "Beta",

se_col = "SE",

effect_allele_col ="EA",

other_allele_col = "NEA",

eaf_col = "EAF",

pval_col = " p Value"

)

exp_LD <- clump_data(exp_dat, clump_r2 = 0.05)

out_dat <- extract_outcome_data(

snps=exp_LD$SNP,

outcomes=' ebi-a-GCST005814',

proxies = TRUE,

maf_threshold = 0.3,

access_token = NULL

)

mydata <- harmonise_data(

exposure_dat=exp_LD,

outcome_dat=out_dat,

action= 1

)

res <- mr(mydata)

res <- generate_odds_ratios(res)

res

het <- mr_heterogeneity(mydata)

het

pleio <- mr_pleiotropy_test(mydata)

pleio

library(MRPRESSO)

run_mr_presso(mydata, NbDistribution = 1000, SignifThreshold = 0.05)

Supplementary Figure S1 Scatter plot for TL and total OA.


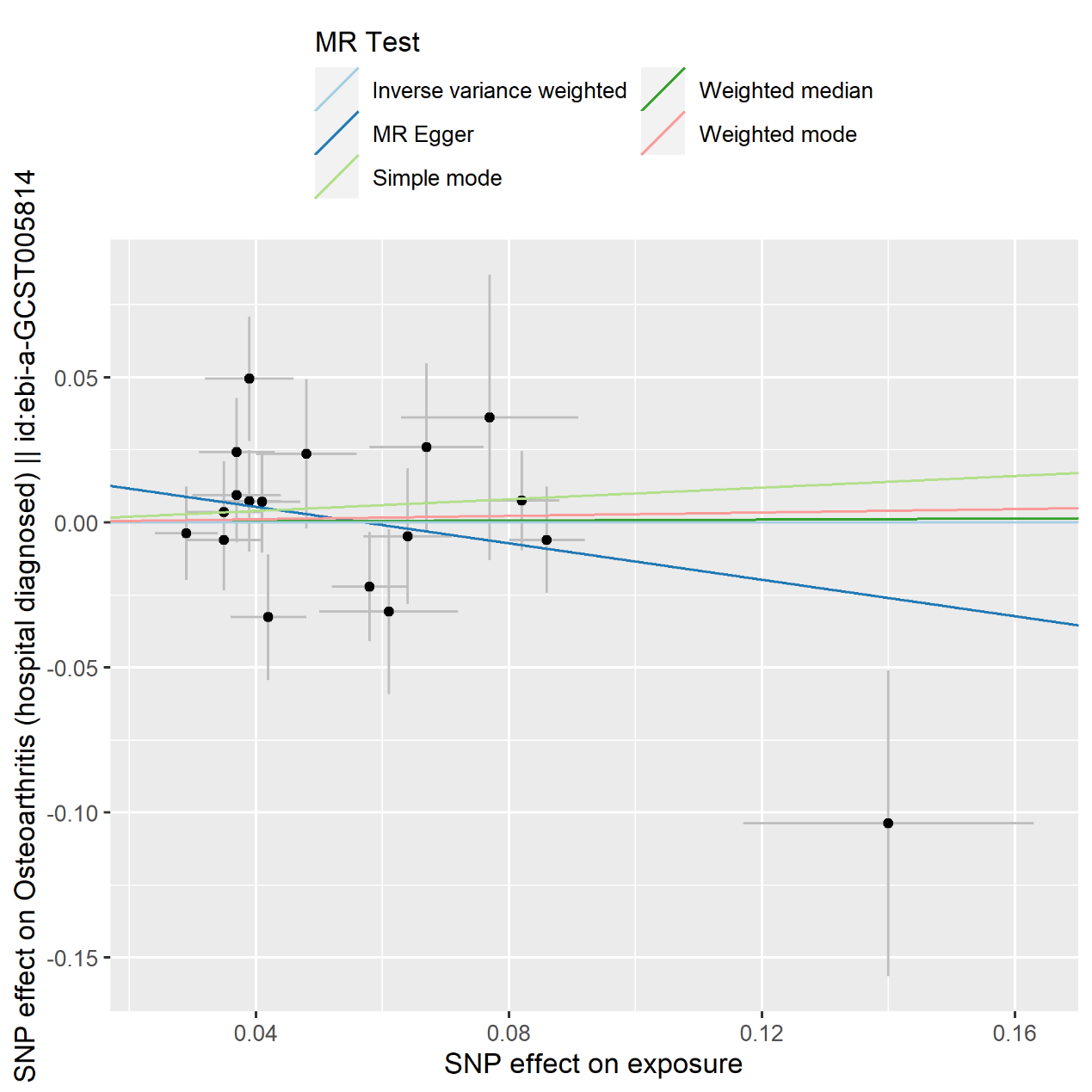


Supplementary Figure S2 Forest plot for TL and total OA.


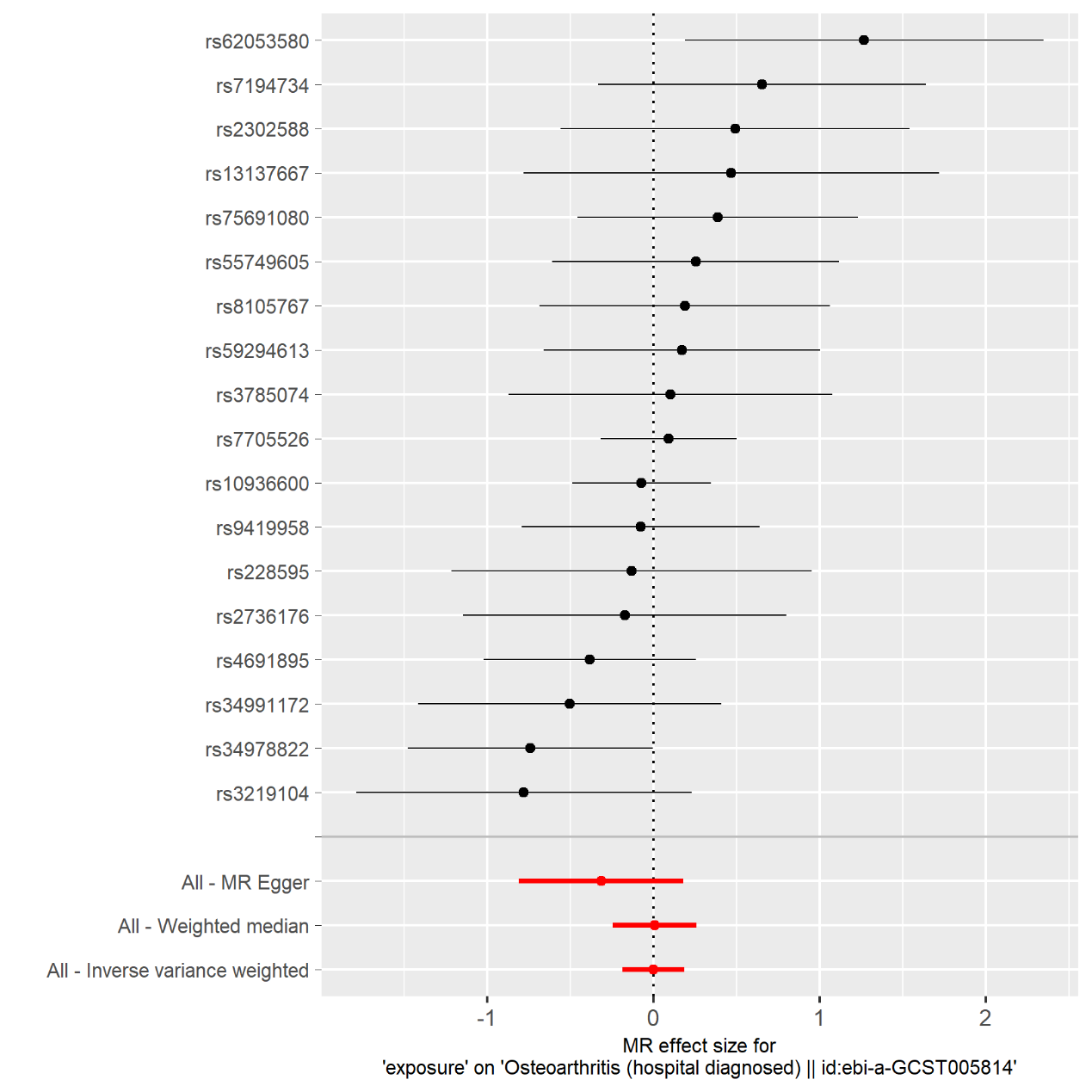


Supplementary Figure S3 Funnel plot for TL and total OA.


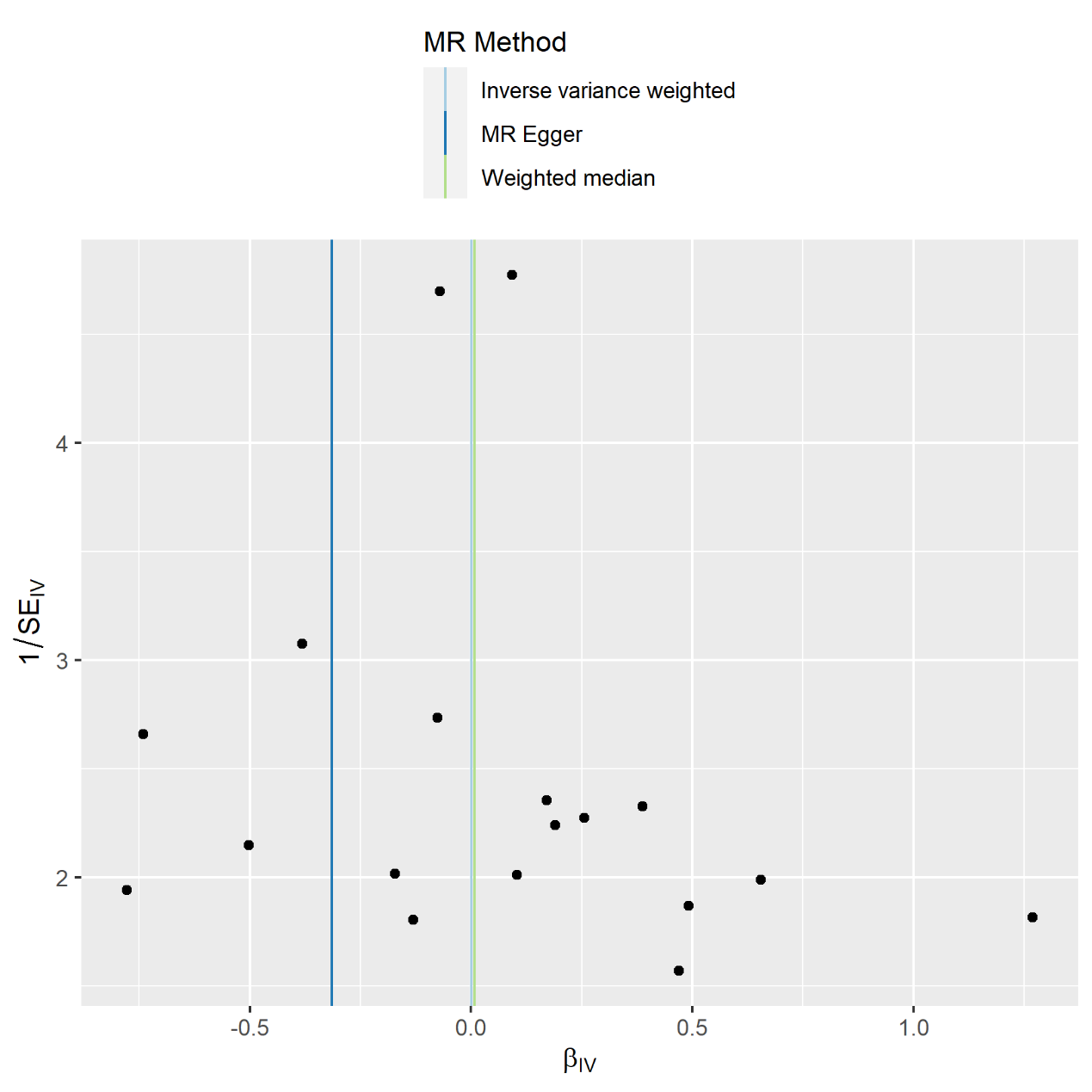


Supplementary Figure S4 Scatter plot for TL and knee OA.


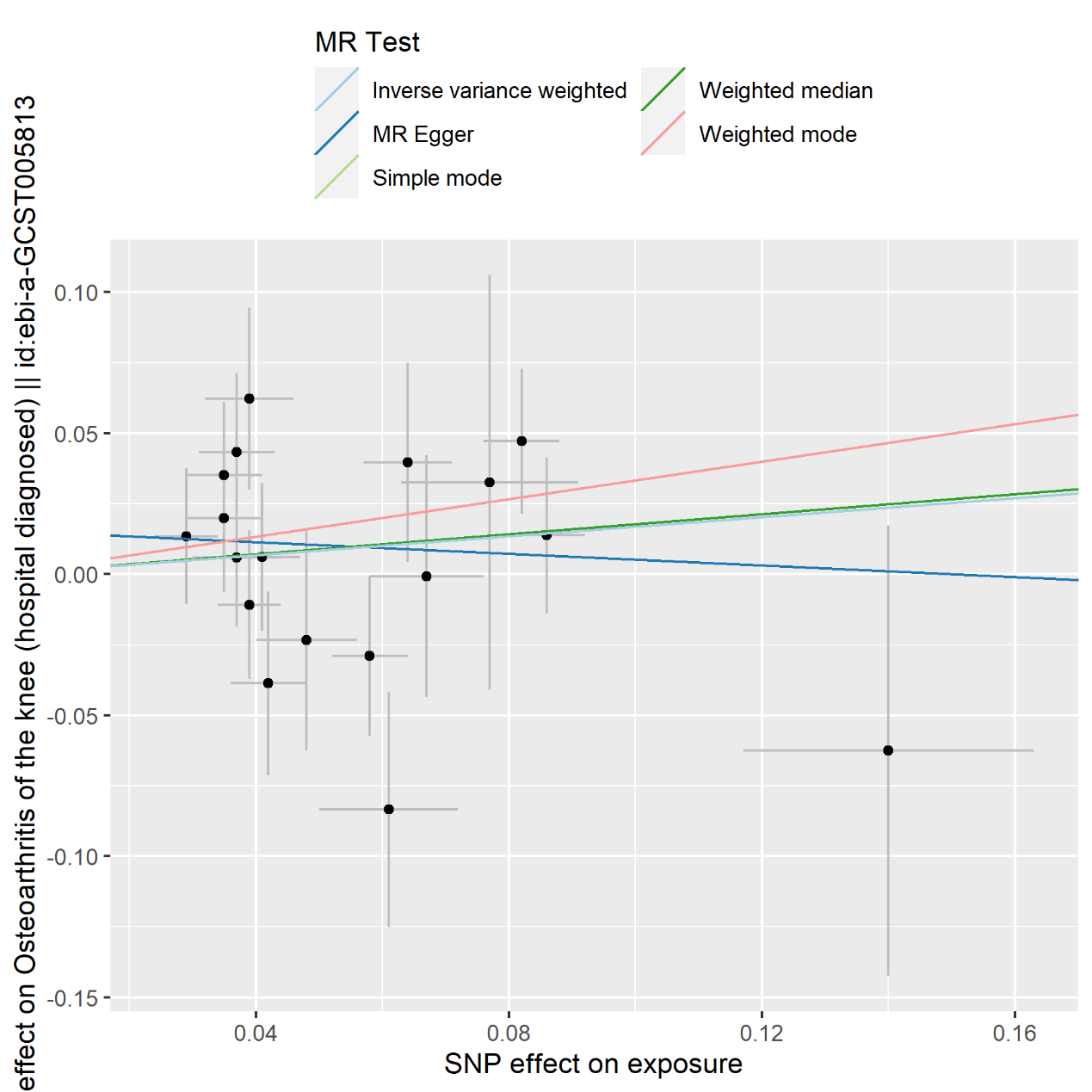


Supplementary Figure S5 Forest plot for TL and knee OA.


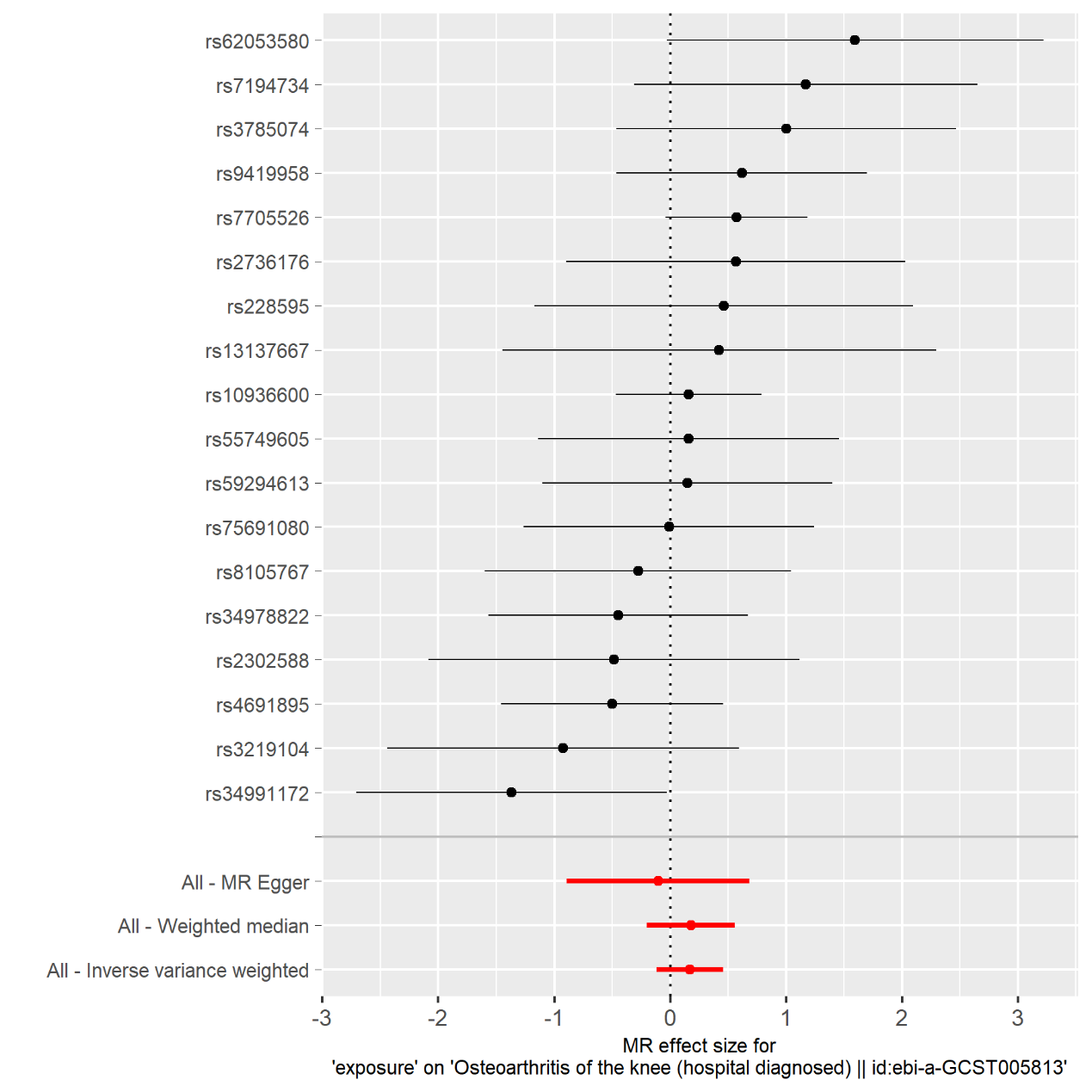


Supplementary Figure S6 Funnel plot for TL and knee OA.


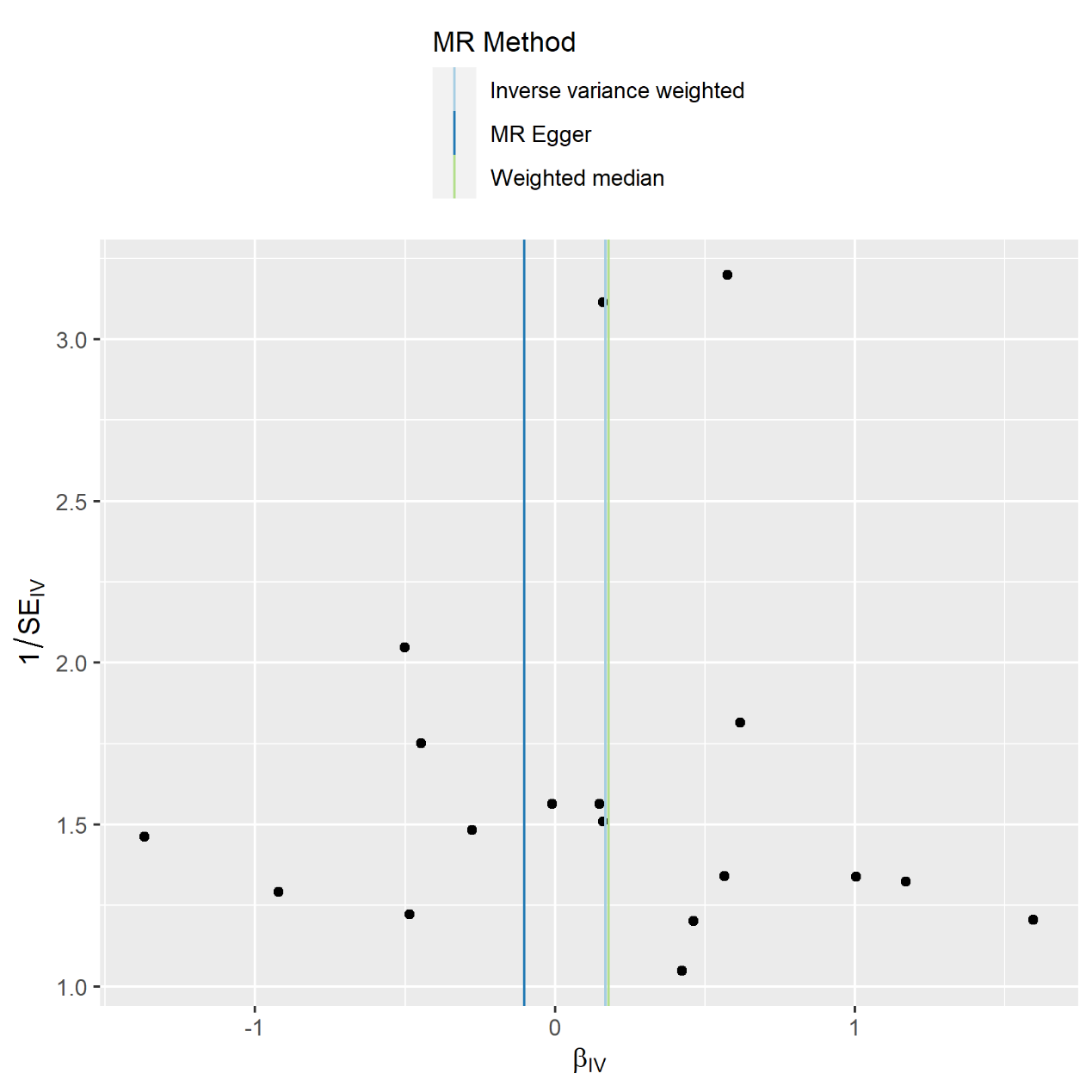


Supplementary Figure S7 Scatter plot for TL and hip OA.


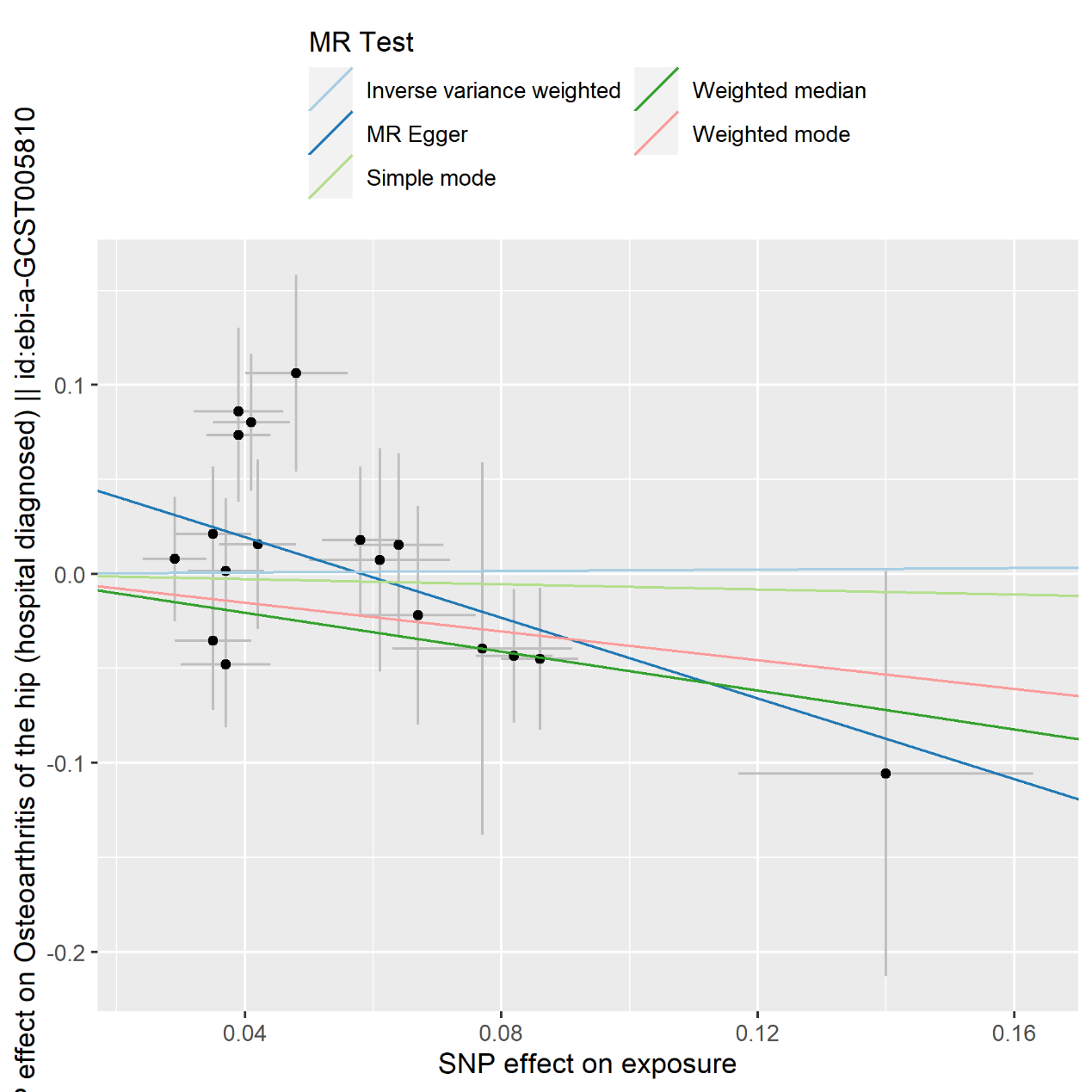


Supplementary Figure S8 Forest plot for TL and hip OA.


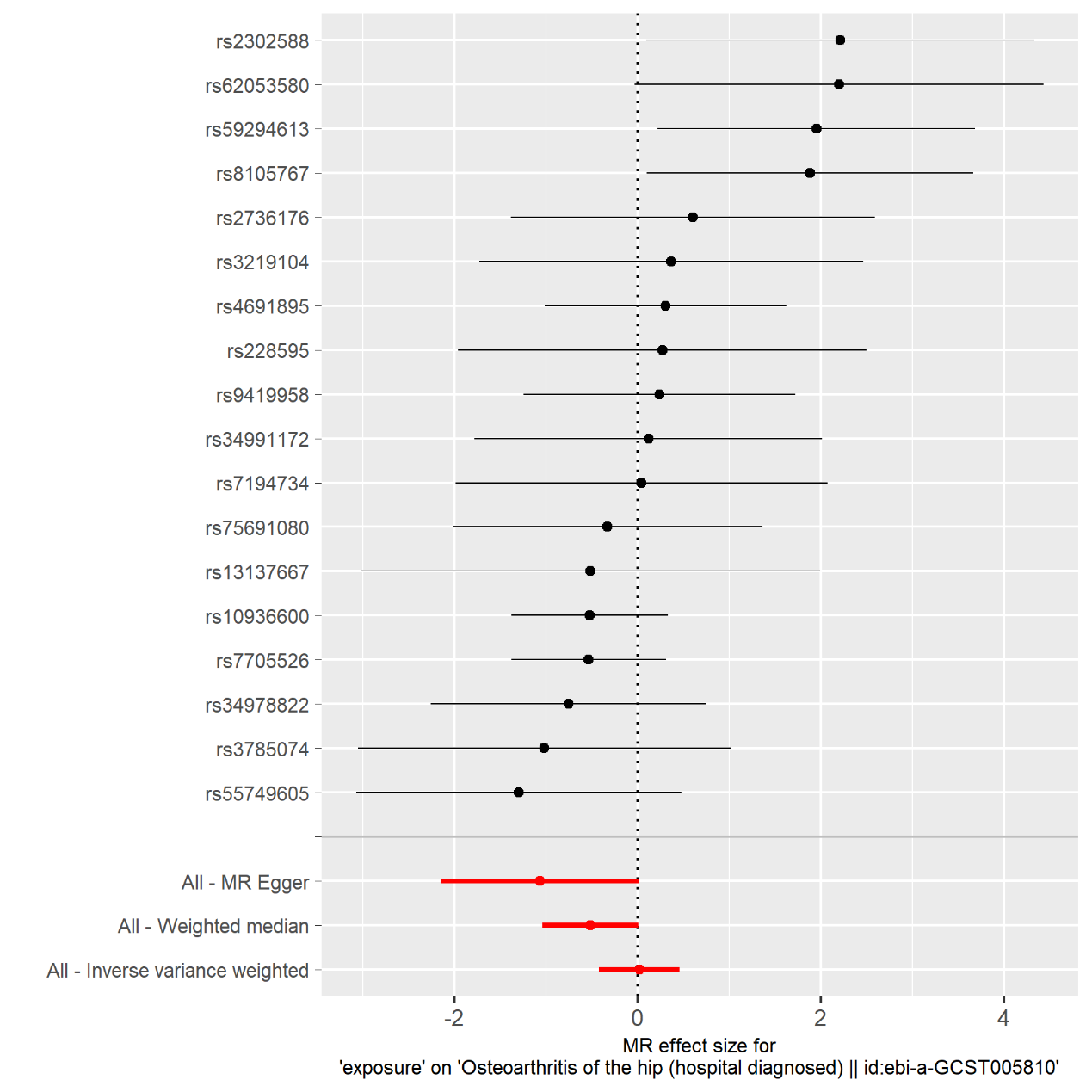


Supplementary Figure S9 Funnel plot for TL and hip OA.


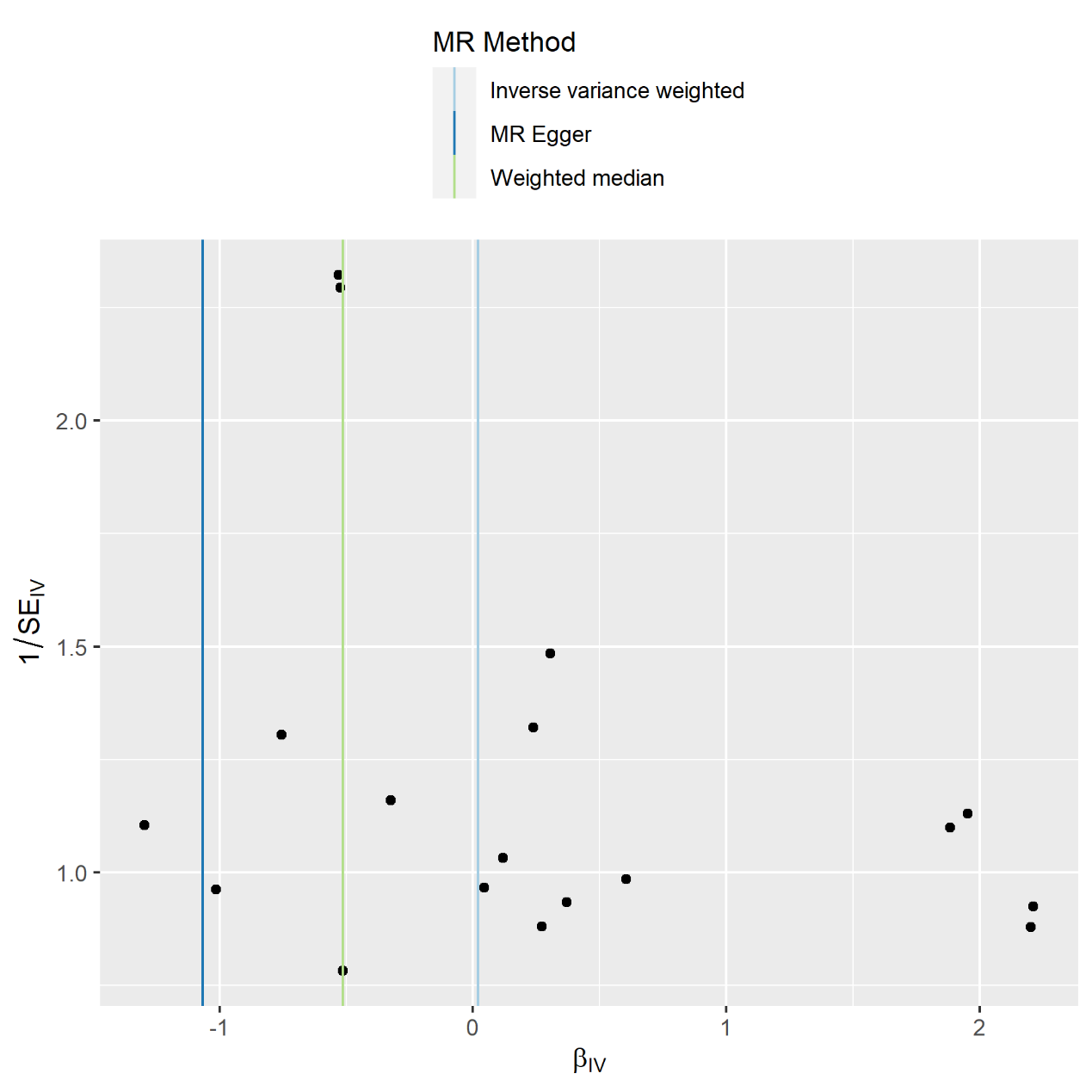


Supplementary Figure S10 Leave-one-out sensitivity analysis for TL on total OA.


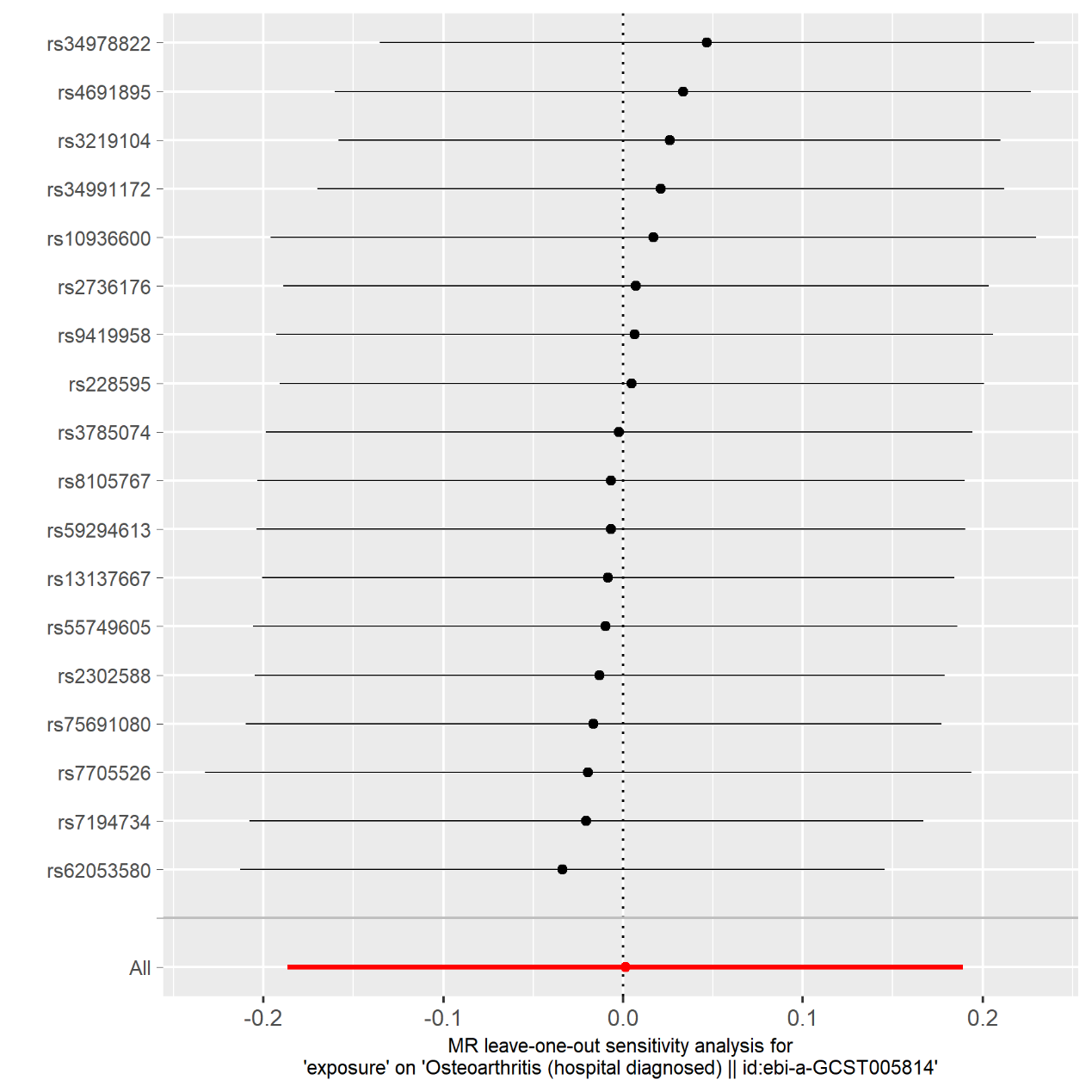


Supplementary Figure S11 Leave-one-out sensitivity analysis for TL on knee OA.


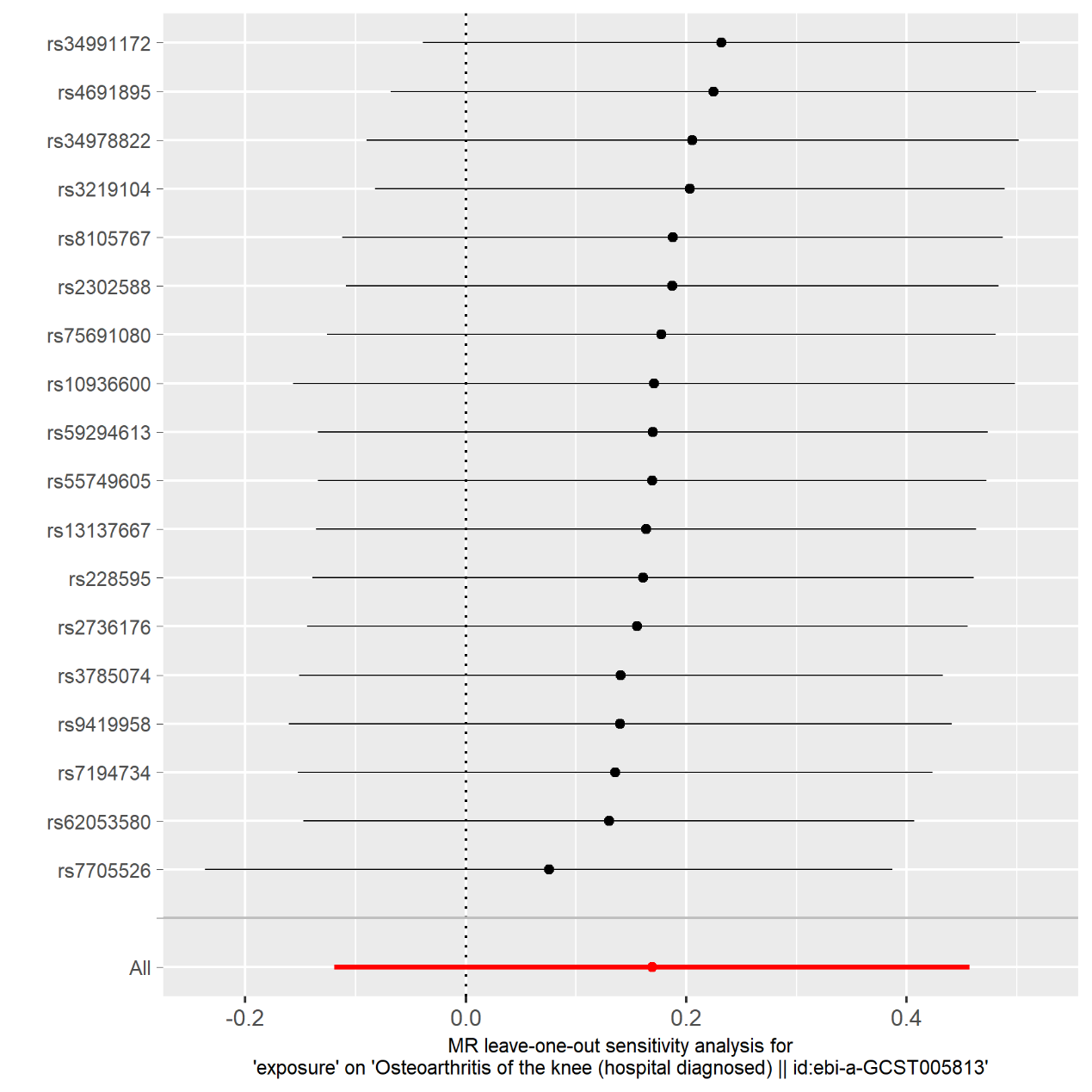


Supplementary Figure S12 Leave-one-out sensitivity analysis for TL on hip OA.


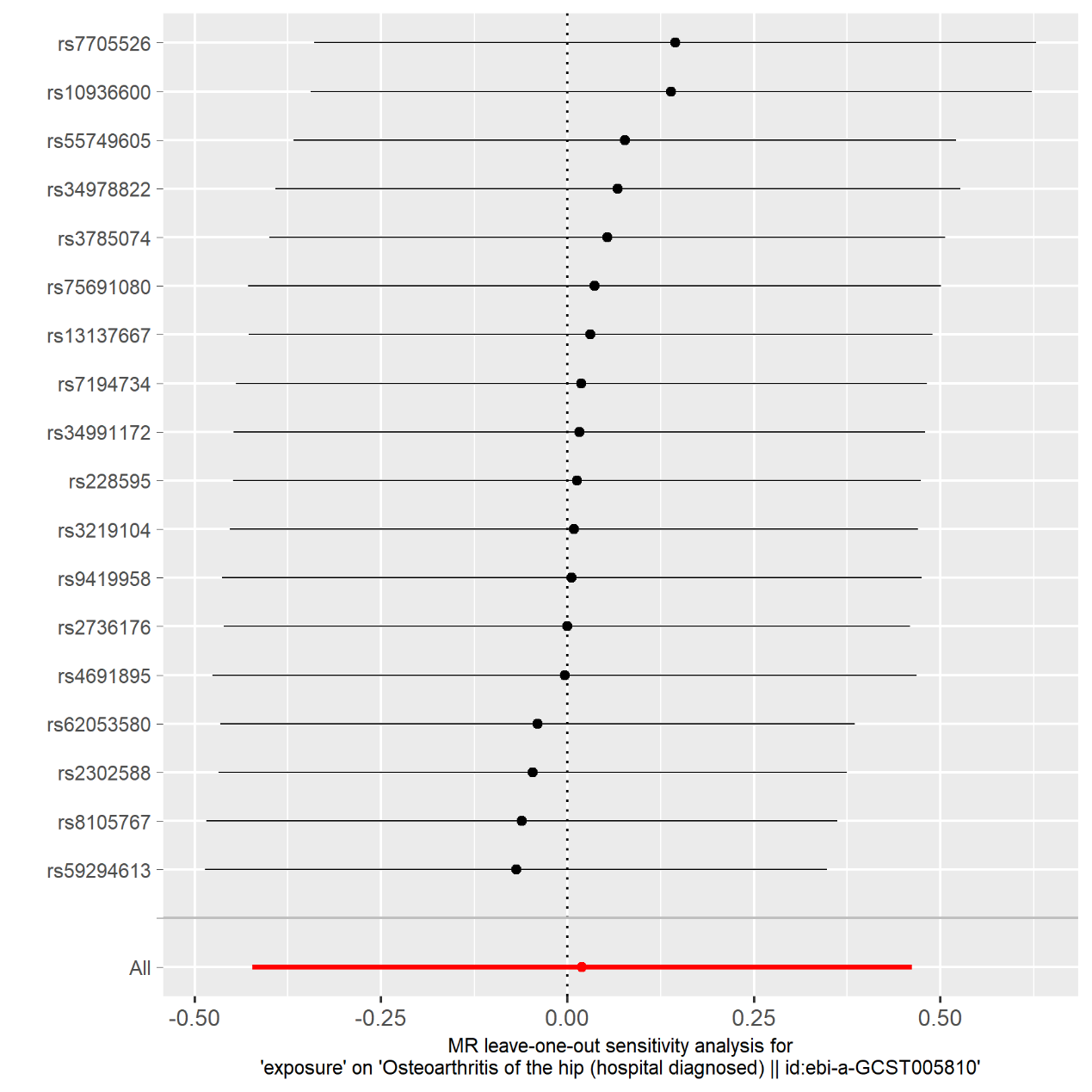

Supplement: Supplementary file 1 [file DataSheet1.docx]
